# Recent Advances in Translational Magnetic Resonance Imaging in Animal Models of Stress and Depression

**DOI:** 10.3389/fncel.2017.00150

**Published:** 2017-05-24

**Authors:** Allison L. McIntosh, Shane Gormley, Leonardo Tozzi, Thomas Frodl, Andrew Harkin

**Affiliations:** ^1^Institute of Neuroscience, Trinity College DublinDublin, Ireland; ^2^Universitätsklinikum A.ö.R, Universitätsklinik für Psychiatrie und Psychotherapie, Medizinische Fakultät, Otto von Guericke UniversitätMagdeburg, Germany; ^3^School of Pharmacy and Pharmaceutical sciences, Trinity College DublinDublin, Ireland

**Keywords:** depression, stress, animal models, MRI, neuroimaging

## Abstract

Magnetic resonance imaging (MRI) is a valuable translational tool that can be used to investigate alterations in brain structure and function in both patients and animal models of disease. Regional changes in brain structure, functional connectivity, and metabolite concentrations have been reported in depressed patients, giving insight into the networks and brain regions involved, however preclinical models are less well characterized. The development of more effective treatments depends upon animal models that best translate to the human condition and animal models may be exploited to assess the molecular and cellular alterations that accompany neuroimaging changes. Recent advances in preclinical imaging have facilitated significant developments within the field, particularly relating to high resolution structural imaging and resting-state functional imaging which are emerging techniques in clinical research. This review aims to bring together the current literature on preclinical neuroimaging in animal models of stress and depression, highlighting promising avenues of research toward understanding the pathological basis of this hugely prevalent disorder.

## Introduction

Major depressive disorder (MDD) is a chronic, heterogeneous disorder with a diverse range of risk factors including genetic predisposition, stress, early life experiences, and environmental factors. MDD often presents with co-morbid anxiety and is linked to several somatic disorders including pain, insomnia, cardiovascular disease, and gastritis. Current antidepressant treatments are inadequate with a delayed onset and a large proportion of patients being treatment resistant. The pathophysiology of MDD remains to be fully elucidated and the development of more effective therapeutics depends on a better understanding of the molecular underpinnings of the disorder. Magnetic resonance imaging (MRI) is a safe, non-invasive technique that can provide valuable information on structural and functional changes in the human brain. MRI is commonly used in patients with MDD to assess changes in brain structure and function relating to symptom severity and treatment response. Alterations in the morphology of brain structures, particularly the hippocampus, have been frequently reported, in addition to differences in microstructural integrity, network connectivity, cerebral blood flow, and metabolite concentrations (Sacher et al., 2012). At present however, the pathophysiology underlying these neuroimaging findings remain unclear which significantly limits the development of novel therapeutic strategies. Preclinical studies in animal models of disease are vital to investigate the molecular and cellular changes that may underpin the neuroimaging hallmarks in MDD. The translation of preclinical research to the clinical setting is often challenging, however the ability to investigate comparable MRI modalities in both humans and animals is particularly valuable. The characterization of animal models of depression by MRI has already provided important insights into potential environmental factors associated with, and physiological mechanisms which may underlie the observed changes in brain structure and function in depressed patients. In addition, investigating neuroimaging markers in animal models allows the examination of molecular and cellular changes post-mortem. Furthermore, MRI in animal models may also provide valuable biomarkers that can be used clinically for treatment response and patient stratification. The advantages and challenges of preclinical neuroimaging as well as methodological concepts have previously been reviewed elsewhere (Hoyer et al., 2014; Jonckers et al., 2015) however a review of the preclinical neuroimaging findings relating to stress and depression is currently lacking. This review aims to address this by discussing the current MRI data in animal models of stress and depression, covering all MRI modalities including structural data, resting-state fMRI, manganese-enhanced MRI, cerebral blood perfusion measurements, and magnetic resonance spectroscopy (MRS). A comparison of the neuroimaging findings in animal models has also been summarized in Table 1.

**Table 1:** **Summary of the structural and functional neuroimaging changes reported in animal models of depression**.

**Animal model**	**Structural MRI**		**Functional MRI**			
	**Volumetric**	**DTI**	**MRS**	**rs-fMRI**	**CBF**	**MEMRI**
Wistar-Kyoto	↓ Hipp ↑ LV ↑ Hipp T2 relaxation time (Gormley et al., 2016)	↓ FA in CC and AC ↑ MD in CC and fornix (Zalsman et al., 2016) Acute stress ↑ MD in Cg and Amyg (Zalsman et al., 2015)		↑ connectivity between Hipp and FC ↓ connectivity between Hipp and SCx, vStr, Cg, LS and CPu in “more immobile” cohort (Williams et al., 2014)	↓ perfusion in the Str and PLC (Gormley et al., 2016)	
Olfactory bulbectomy (OB)	↓ T2 relaxation times in Hipp, VCx and RSc (Gigliucci et al., 2014)					
Interferon alpha (IFN-α)						↓ pituitary volume ↓ activation in VCx and sensory cortices (Daducci et al., 2014)
Chronic mild stress (CMS)	↑ LV No change in Hipp (Henckens et al., 2015) No change in Hipp volume but subtle change in morphology (Delgado y Palacios et al., 2011) ↓ Hipp volume (Lee et al., 2009) *Ex vivo* ↑ VTA, BNST and DRN ↓ Cg and NAcc linked to social avoidance (Anacker et al., 2016)	↑ MD in FC, Hipp, Hypo, CPu and CC ↑ RD and AD in FC and ↑ AD in Hypo (Hemanth Kumar et al., 2014) ↓ MK and RK in Hipp and ↑ AD in CPu (Delgado y Palacios et al., 2014) ↑ RD in Amyg ↓ MK in CPu of anhedonic-like compared to resilient (Delgado y Palacios et al., 2011) ↑ RD in FC and Hypo (Hemanth Kumar et al., 2014)	↓ Glu in dHipp (Hemanth Kumar et al., 2012) Maternal stress ↓ Glu in dHipp of offspring (Huang et al., 2016) Chronic FST ↓ Glu in PFC and Hipp (Li et al., 2008) ↓ Glx in PFC (Hemanth Kumar et al., 2012) ↑Glu in vHipp (Delgado y Palacios et al., 2011) ↑ Cho in PFC (Grandjean et al., 2016) ↑ Cho in Hipp and Amyg following single restraint stress (Han et al., 2015) ↑ mI in Hipp (Hemanth Kumar et al., 2012) ↑ mI in Amyg (Grandjean et al., 2016) Acute FST ↑ GABA in Hipp of CMS-offspring (Huang et al., 2016) *Ex vivo* ↑ GABA in Cg and PFC (Perrine et al., 2014; Drouet et al., 2015) ↓ NAA in Hipp (Li et al., 2008; Xi et al., 2011; Hemanth Kumar et al., 2012) ↑ NAA in Amyg and Hipp following single restraint stress (Han et al., 2015)	↑ connectivity within Cg and sensory networks ↑ connectivity between PFC and both Amyg and Pir ↑ vHipp and Amyg connectivity ↑ connectivity between Cg and both Pir and Amyg (Grandjean et al., 2016) ↑ connectivity within DMN (Henckens et al., 2015)		↓ connectivity between DRN and SN, Hipp, EC and IC, and ↑ connectivity with MSN (Gordon and Goelman, 2016)
Congenital learned helplessness (cLH)			↓ Glu in dHipp ↓ Cho in Hipp and FC (Schulz et al., 2013)	↑ connectivity between DRN and SCx, OC, FC and CPu ↑ connectivity between vHipp and RSc and CPu ↑connectivity between RSc and Cg (Gass et al., 2014a) ↓ inter-hemispheric connectivity in sensory, motor, Cg and ILC and NAcc and DRN (Ben-Shimol et al., 2015)	↓rCBV in left Hab and ↑ rCBV in BNST (Gass et al., 2014a)	
5-HT_1A_ receptor knockout mice				↓ connectivity between PFC, RSc and EC and the Hipp (CA1 and DG) (Razoux et al., 2013)		

*AC, anterior commissure; AD, axial diffusion; Amyg, amygdala; BNST, bed nucleus of the stria terminalis; CBF, cerebral blood flow; CC, corpus callosum; Cg, cingulate cortex; Cho, Choline; CPu, caudate putamen; dHipp, dorsal hippocampus; DG, dentate gyrus of the hippocampus; DRN, dorsal raphe nucleus; DTI, diffusion tensor imaging; EC, entorhinal cortex; FA, fractional anisotropy; FC, frontal cortex; FST, forced swim test; GABA, γ-aminobutyric acid; Glu, gliutamate; Glx, combined glutamate and glutamine; Hab, habenula; Hipp, hippocampus; Hypo, hypothalamus; IC, insular cortex; ILC, infralimbic cortex; LS, lateral septum; LV, lateral ventricles; MD, mean diffusion; MEMRI, manganese-enhanced magnetic resonance imaging; mI, myo-inositol; MK, mean kurtosis; MRS, magnetic resonance spectroscopy; MSN, medial septal nucleus; NAA, N-acetylaspartic acid; NAcc, nucleus accumbens; OC, orbital cortex; PFC, prefrontal cortex; PLC, pre-limbic cortex; Pir, piriform cortex; rCBV, regional cerebral blood volume; RD, radial diffusion; RK, radial kurtosis; RSc, retrosplenial cortex; rs-fMRI, resting-state functional magnetic resonance imaging; SCx, somatosensory cortex; SN, substantia nigra; VCx, visual cortex; vHipp, ventral hippocampus; VTA, ventral tegmental area*.

## Animal models of depression

Animal models of depression are important to screen novel compounds for antidepressant action, and to investigate neurochemical and physiological processes that may underlie depressive behaviors. Modeling depressive disorders in rodents is challenging, particularly as many of the symptoms cannot be convincingly determined in rodents. Furthermore, as the pathophysiological processes and genetic risk factors underlying the human disorder and the mechanism of antidepressant action are currently not well understood, developing valid models is problematic. Animal models are therefore unlikely to mirror the full extent of the disorder, however they can be validated through three sets of criteria: face, construct, and predictive validity. The model should therefore reflect features of the human condition (face validity), have some relevance to the etiological basis of the disorder (construct validity) and finally respond similarly to treatments (predictive validity). Animal models can therefore be a useful tool to investigate the underlying pathophysiology or potential of novel therapeutics.

Several techniques have been used to induce depressive-like symptoms in animals including acute exposure to stressors as well as more chronic interventions that reflect aspects of depression etiology such as stress or genetic manipulation. The forced swim test (FST) is a paradigm based on the observation that animals who are faced with an inescapable stressor will desist from attempting to escape after a period of time. Once a rodent is placed in a large container of water from which they cannot escape they will initially vigorously attempt to escape, but will then cease escape orientated behaviors and the immobility seen in the FST has been reported to reflect “behavioral despair.” Antidepressant treatments have been shown to reduce immobility time and therefore the FST has been used as a rapid screen for antidepressant activity in novel compounds. The FST test however has very little construct validity for depression as it does not have any etiological relevance to the disorder. Therefore, more relevant interventions have been developed to induce a depressive-like phenotype in animals and the FST is used as a behavioral measure in these. A well characterized model for depression is the olfactory bulbectomized (OB) rat which involves bilateral removal of the olfactory bulbs, resulting in retrograde degeneration of neurons that project from the olfactory bulbs and loss of efferent connections to sub-cortical brain structures. This, combined with loss of olfaction which may constitute a profound stressor, produces a depressive-like and highly agitated behavioral phenotype which is associated with alterations in endocrine and neurotransmitter levels similar to those reported in depressed human patients (for review see, Kelly et al., 1997; Harkin et al., 2003; Song and Leonard, 2005). The OB model also has good predictive validity as OB-induced hyperactivity in the open field, indicative of a highly anxiety-like phenotype, is sensitive to chronic, but not acute antidepressant treatment. Chronic mild stress (CMS) is also commonly used to induce depressive-like phenotype in animals and has better construct validity than the OB rat. Although the exact protocol can vary, in general CMS involves exposing rodents to several stressors of different intensity and duration over a period of several days and induces a robust depressive-like behavioral phenotype. Several inbred rat strains have been proposed as models of genetic vulnerability to depression, including the Wistar-Kyoto (WKY) rat and also the congenital learned helpless (cLH) rat. Originally used as a normotensive control for the spontaneously hypertensive rat (SHR), the Wistar-Kyoto rat strain endogenously expresses some of the behavioral, endocrine, and neurotransmitter changes found in depressed human patients (Redei et al., 2001). Similarly, the cLH rats demonstrate congenital learned helpless behavior and anhedonia, predictive validity with antidepressant treatments and biochemical changes including alterations in hippocampal glutamatergic systems. Genetic manipulations have also been used to investigate the alterations and/or underlying mechanisms associated with stress, anxiety, and depressive-like symptoms. Given the fact that serotoninergic dysfunction is implicated in stress and depression with many of the current antidepressants modulating serotonin activity, studies have used genetic knockouts of serotonin receptors to further elucidate the effects of serotonin dysregulation (Savitz et al., 2009). The serotonin 5-HT1A receptor knockout (5-HT_1A_-R KO) mouse, although not considered a model of depression, is one such manipulation used to investigate the neural circuits affected by altered serotonin transmission.

All of these models demonstrate some face, construct, and predictive validity for depression and using translational approaches such as MRI may serve to increase our understanding of the physiological basis of the functional and structural brain alterations observed in depressed patients by allowing examination of molecular and cellular alterations post-mortem.

## MRI in animal models of depression

### Structural changes

#### Volumetric MRI

Recent meta-analyses of structural imaging studies in the clinical literature have detected volumetric changes in a number of brain regions in MDD relative to controls. The most robust finding in the literature is reduced hippocampal volume in patients (Campbell et al., 2004; Videbech, 2004; Kempton et al., 2011; Arnone et al., 2012; Schmaal et al., 2016), but some reports have highlighted a decrease in the volume of prefrontal, dorsomedialprefrontal, orbitofrontal and cingulate cortices, and striatum (Arnone et al., 2012; Bora et al., 2012; Sacher et al., 2012; Lai, 2013) as well as increased volume in the lateral ventricles (Kempton et al., 2011).

Although hippocampal volume loss might be dependent on patient age and disease duration (McKinnon et al., 2009; Schmaal et al., 2016), a meta-analysis of studies in first episode patients revealed significant hippocampal volume reductions, suggesting that smaller hippocampal volume may be a possible risk factor for depression, rather than a marker of disease progression (Cole et al., 2011). This is in agreement with a 3-year longitudinal study, which reported no significant reduction in hippocampal volume of patients, but did show that smaller baseline volume was associated with poorer clinical outcome (Frodl et al., 2008a). It should be noted that, following a whole brain voxel based morphometry analysis, a decrease in gray matter density in the hippocampus in patients with ongoing depression over a 3 year period was detected, thus highlighting a potential impact of the method of analysis on the findings (Frodl et al., 2008b).

Changes in hippocampal volume also appear to be sensitive to antidepressant treatment, showing an increase in volume following traditional antidepressant (Frodl et al., 2008a) and electroconvulsive (ECT; Abbott et al., 2014) therapies. Fu et al. (2013) suggested that lower right hippocampal volume could even be a predictor of poor antidepressant response. Reduced subgenual anterior cingulate cortex volume persists despite successful treatment with antidepressant drugs (Drevets et al., 1997) but chronic lithium treatment, which exerts robust neurotrophic effects in animal models, has been associated with an increase in gray mater volume in treatment responders in this and other prefrontal areas (Drevets et al., 2008; Moore et al., 2009).

Interestingly, smaller hippocampi in depressed patients have been found to be associated with increased markers of glucocorticoid receptor activation in peripheral plasma, suggesting that gray matter loss in this structure might be related to changes in HPA axis function (Frodl et al., 2012).

Translational neuroimaging studies have reported structural alterations in the rodent brain in animal models of depression. The Wistar-kyoto (WKY) rat is an inbred rat strain with an inherent anxiety- and depressive-like behavioral phenotype. *In vivo* MRI has shown increased lateral ventricular volume and decreased hippocampal volume in adult WKY rats compared to the Wistar comparison strain with no change in total cortical volume (Gormley et al., 2016). Alterations in T1 and T2 relaxation times can provide information about tissue characteristics and increased T2 relaxation times were evident in the hippocampus of WKY rats compared to Wistar controls (Gormley et al., 2016), a finding that may be linked to oedema or changes in vascular permeability. Work in our group has also investigated MRI changes in the olfactory bulbectomized rat (OB) and showed opposite changes with reductions in T2 relaxation times in the hippocampus, visual cortex and left retrosplenial cortex (Gigliucci et al., 2014). This highlights the heterogeneity of animal models and may indicate alterations in relaxation times could be linked to specific phenotypes. Furthermore, this emphasizes the importance of selecting an appropriate animal model as the OB rat may be more related to anxiety rather than depressive-like behaviors. Structural analysis revealed a trend toward increased ventricular volume in OB rats however no changes in hippocampal volume were observed (Gigliucci et al., 2014). Reductions in T2 relaxation times have been linked to microglial activation and also increased cell packing density in the brain parenchyma (Ding et al., 2008; Blau et al., 2012). Given the lack of overt inflammation, it is most likely that changes in cell density or morphology may account for the decreased T2 relaxation times reported in the OB rats.

Reduced hippocampal volume has also been linked to anxiety-like behavior, with mice bred for high anxiety showing reduced hippocampal volume compared to those bred for low anxiety traits (Kalisch et al., 2006). The structural changes may also be linked to the depressive-like behavioral phenotype observed in these animals, however the authors show that in control animals, hippocampal volume positively correlated with anxiety-like but not depressive-like behavior and therefore suggest the differences in hippocampal volume are primarily linked to anxiety-like traits.

Chronic stress paradigms have also been shown to produce changes in rodent brain morphology. Structural MRI of post-mortem brains of Wistar rats that were subjected to a 10 day immobilization stress, have shown an increase in lateral ventricular volume without any change in hippocampal volume or morphology when compared to non-stressed controls (Henckens et al., 2015). Delgado y Palacios et al. (2011) also report no change in hippocampal volume in rats exposed to 8 weeks of CMS, although subtle changes in hippocampal morphology were evident. In contrast, Lee et al. (2009) report that 21 days of immobilization stress in rats results in a 3% reduction in baseline hippocampal volume, assessed by longitudinal MRI, a change which was not observed in control animals. There was no change observed in anterior cingulate or retrosplenial cortical volumes reported (Lee et al., 2009). The contradictory reports relating to hippocampal volume may be related to stressor severity and/or the *ex vivo* acquisition of structural data. *Ex vivo* MRI on post-mortem brains following 10 days of social defeat in mice shows that the volume of the ventral tegmental area (VTA), bed nucleus of the stria terminalis (BNST) and the dorsal raphe nucleus (DRN) positively correlate with social avoidance scores in animals, indicating that these areas may be linked to stress vulnerability (Anacker et al., 2016). Social avoidance scores also negatively correlated with size of both the cingulate cortex and nucleus accumbens suggesting decreased volume in stress-sensitive mice. Interestingly DTI metrics largely correlated in opposite directions to volumetric changes indicating increased volume is accompanied by reduced diffusion in the brain regions investigated.

Volumetric alterations have also been associated with antidepressant effects in offspring following administration of a ketogenic (high ketone) diet (KD) during pregnancy. Whilst small structural changes were observed at weaning (PND 21.5), KD offspring showed increased frontal cortical and cerebellar volume, but interestingly, decreased volume of the hippocampus, striatum, and some cortical areas in adulthood (Sussman et al., 2015). The observed decreased hippocampal volume is somewhat counterintuitive to an antidepressant effect given that reductions in hippocampal volume are associated with depressive symptoms. The authors suggest this may be due to reduced dendritic branching or abnormal neuronal differentiation resulting from low protein consumption however as this is a single study examining MRI related KD effects, further work is required to comprehensively assess the long-term effects of KD diet on behavior and brain structure.

Together these data suggest that increased lateral ventricular volume and decreased hippocampal volume are relatively consistent hallmarks in both patients with MDD and animal models, adding to their face validity. Although contrasting findings may be due to study design, analysis method or method of acquisition they could also reflect subtypes of stress/depression and the diversity of patient populations that may be modeled in animals.

#### Diffusion imaging

Diffusion tensor imaging (DTI) allows for an assessment of *in vivo* white matter microstructural integrity through measurement of the restriction in diffusion of water molecules in brain tissue (Moser et al., 2009). Several DTI studies in patients with unipolar depression have reported decreased cortical fractional anisotropy (FA), a measure of white matter integrity and fiber directionality in the frontal and temporal lobes (for review see Sexton et al., 2009) as well as in the amygdala (Arnold et al., 2012). This seems to be evident in both late life depression (Bae et al., 2006; Nobuhara et al., 2006) and in young adults (Li et al., 2007). Data suggests that altered FA values may be a marker of pre-disposition to depression or may occur early in the course of disease, as patients with first onset depression have been shown to exhibit decreased FA values in frontal cortical regions such as the anterior cingulate cortex (Zhu et al., 2011) and the frontal gyrus (Ma et al., 2007).

Currently, tractography studies show that first episode depressed patients exhibit increased cortico-limbic structural connectivity (Fang et al., 2012) and that depression is associated with a disruption in the pathways linking subcortical regions, including the hippocampus, to the prefrontal cortex (Liao et al., 2013) as well as alterations in the superior longitudinal and fronto-occipital fascicule (Murphy and Frodl, 2011). Finally, preliminary results from the largest study to date have also highlighted alterations in the corpus callosum of MDD subjects (Kelly et al., 2016).

DTI, based on Gaussian distribution of water molecules, and more recently diffusion kurtosis imaging (DKI) which attempts to account for biological variation in water diffusion and provide a more accurate model of diffusion, have both identified alterations in microstructural integrity in animal models of depression. Common metrics reported in DTI and DKI studies are mean diffusion or kurtosis across all directions (MD or MK), radial diffusion or kurtosis (RD or RK; movement in the transverse direction) and axial diffusion or kurtosis (AD or AK; movement along the main axis of diffusion). Although, interpretation of diffusion imaging has not been fully explored, microstructural organization of the brain, including cellular membranes, myelinated axons, intracellular organelles, and dendritic architecture, affects the orientation of water diffusion.

DTI and DKI studies have identified alterations in the microstructural integrity of gray matter regions in rats exposed to CMS. Increased mean diffusion values in the bilateral frontal cortex, hippocampus, hypothalamus, caudate putamen and corpus callosum were reported in rats exposed to CMS compared to control animals, which may relate to reduced membrane density or tissue degeneration (Hemanth Kumar et al., 2014). This was accompanied by increased radial and axial diffusion in the frontal cortex and increased AD in the hypothalamus. It is suggested that increased RD may be due to demyelination while the AD alterations may be attributed to axonal loss. Conversely, decreased mean and radial kurtosis (magnitude of restrictions perpendicular to the principle direction of diffusion) has been reported in the hippocampus of animals exposed to CMS (Delgado y Palacios et al., 2011) irrespective of sensitivity to the stress protocol. Further work by the same group expanded the neuroimaging markers relating to stress sensitivity and resilience. Specifically, increased axial diffusion in the caudate putamen (CPu) demonstrates microstructural alterations within the CPu (Delgado y Palacios et al., 2014). Furthermore, increases in radial diffusion were observed in the amygdala (Delgado y Palacios et al., 2011) as well as frontal cortex and hypothalamus (Hemanth Kumar et al., 2014) of CMS animals. This may reflect dendritic atrophy, augmented arborization or demyelination as alterations in hippocampal kurtosis parameters were accompanied by decreased staining of microtubule associated protein 2, which is involved in stabilization of neuronal cytoarchitecture (Delgado y Palacios et al., 2011). In addition, by subdividing the CMS group into those sensitive or resilient to CMS Delgado et al. showed decreased mean kurtosis values in the caudate putamen of animals exhibiting stress-induced behavioral anhedonia compared to stress-resilient counterparts. Such alterations in diffusion metrics may be attributed to differences in dendritic arborization or potential alterations in microglial morphology due to activation states. Although, further studies are required to elucidate the molecular and cellular mechanisms underlying these microstructural changes, these data highlight the CPu as a region altered by stress and potentially valuable in identifying individuals vulnerable to stress. Furthermore, this highlights the importance of analyzing cohorts based on their behavioral responses to stressors as failure to identify possible “resilient” subgroups may result in misleading conclusions.

The effect of stress on tissue architecture in both control and stress sensitive WKY rats has also been investigated. WKY rats show decreased FA in the corpus callosum and anterior commissures and increased mean diffusion in both the fornix and corpus callosum compared to the Wistar comparator strain (Zalsman et al., 2016). Furthermore, exposing WKY rats to early-life stress produced significantly higher mean diffusion values compared to control in the amygdala, cingulate cortex and external capsule. Conversely, early stress decreased mean diffusion in the cingulate cortex, amygdala, and external capsule in Wistar rats (Zalsman et al., 2015). This suggests that stress alters the structural integrity of brain areas linked to emotional regulation differently in animals with genetic vulnerability.

DTI studies in depressed patients have shown decreased fractional anisotropy in the frontal cortex, cingulate and corpus callosum, changes which are mirrored in the preclinical research supporting the translational relevance of these measures. Interestingly, diffusion kurtosis imaging is not widely used in clinical neuroimaging, however preclinical results from CMS studies suggest kurtosis parameters, particularly in the caudate putamen, may be useful in identifying susceptible from resilient phenotypes and highlights the importance of animal models.

### Functional MRI

#### Magnetic resonance spectroscopy

Throughout the years, the investigation of the biochemical alterations associated with MDD has highlighted changes in neurotransmitter systems of depressed patients (Crow et al., 1984; Cheetham et al., 1989; Yildiz-Yesiloglu and Ankerst, 2006; Rajkowska and Stockmeier, 2013). In particular, proton MRS is a non-invasive technique that has been often used to detect differences in tissue concentration of various chemical compounds *in vivo* (Jansen et al., 2006). Even on routinely available 1.5 T scanners, this technique can identify N-acetylaspartate (NAA), choline (Cho), and creatine (Cr). At higher field strengths (3-7 T), glutamate (Glu), glutamine (Gln) and myoinositol (Ins), can also be individually resolved. Finally, γ-Aminobutyric acid (GABA) as well as glutathione (GSH) are optimally quantified at the highest field strengths with special editing and acquisition techniques (Bustillo, 2013; Rae, 2014), although GSH has been shown to be reliably measured at lower fields using fitting routines such as LCModel after acquisition (Terpstra et al., 2003).

MRS studies have often reported a reduction of glutamate, the most common excitatory neurotransmitter, in the prefrontal cortex of MDD compared to controls (Yildiz-Yesiloglu and Ankerst, 2006; Luykx et al., 2012; Miladinovic et al., 2015). Glutamine (Gln), an amino acid synthetized from glutamate in the glia (Martinez-Hernandez et al., 1977), and GABA have also been found to be decreased in MDD in this region (Walter et al., 2009; Pehrson and Sanchez, 2015). Glutathione, which is involved in synthesis and degradation of proteins and DNA (Maher, 2005), has recently been found to be reduced in the occipital cortex of depressed patients, suggesting reduced anti-oxidative capacity in this cohort (Godlewska et al., 2015). Choline, which is related to membrane metabolism and turnover, has been found to correlate with mood in healthy subjects (Jung et al., 2002) and to be increased in MDD patients compared to healthy controls in the basal ganglia (Yildiz-Yesiloglu and Ankerst, 2006). Most studies have not shown any difference in NAA levels in depressed patients (Yildiz-Yesiloglu and Ankerst, 2006) and inconsistent results for Cr levels (Venkatraman et al., 2009). Thus, the most consistent findings in MDD are decreased excitatory and inhibitory neurotransmitters as well as glutathione, and elevated Ch levels in some regions, indicative of increased membrane phospholipid turnover.

The low signal to noise ratio and large voxel size required has hindered the progression of preclinical MRS studies, however with greater sensitivity achieved with magnetic fields as high as 11.7T currently available, several recent studies have shown alterations in a range of metabolites in animal models of depression.

Exposing the offspring of dams subjected to CMS to an acute stressor in adolescence (a single forced swim test) decreased glutamate MR signal in the right hippocampus (Huang et al., 2016). Similarly, chronic exposure to the FST has been reported to decrease the glutamate signal in both the prefrontal cortex and hippocampus in rats (Li et al., 2008). Reductions in the combined glutamate and glutamine signal have also been reported in the prefrontal cortex of mice exposed to CMS (Hemanth Kumar et al., 2012). In contrast, Delgado y Palacios et al. (2011) report increased hippocampal glutamate levels in animals susceptible to CMS compared to both resilient and control groups. These conflicting changes in hippocampal glutamate may be due to the type of stressor used (acute compared to chronic) or regional differences within the hippocampus. Delgado et al. positioned a 2 mm^3^ voxel in the ventral part of the left hippocampus whereas Huang et al. (2016) positioned their 2.5 × 4 × 4 mm voxel in the dorsal area of the right hippocampus. Similar decreases in dorsal hippocampal glutamate have been reported in rats bred for learned helplessness (cLH) compared to Sprague-Dawley controls and also following CMS (Hemanth Kumar et al., 2012; Schulz et al., 2013).

Schulz et al. also report reductions in choline in both the hippocampus and frontal cortex (encompassing the cingulated and motor cortex) of cLH rats (Schulz et al., 2013). Conversely, a recent study reported increased choline levels in the prefrontal cortex following chronic psychosocial stress (Grandjean et al., 2014), which may reflect differences in preclinical models used (genetic vs. stress) or regional differences between the frontal and prefrontal cortex. Alterations in choline levels have also been shown by Han et al. with increased levels in both the hippocampus and amygdala following a single prolonged restraint stress (Han et al., 2015). Choline has also been shown to be sensitive to antidepressant treatments as Sartorius et al. (2003) showed electroconvulsive stimulation (ECS), the preclinical model of ECT, increased choline levels in the hippocampus of normal animals. Similarly, ECS increased the absolute concentrations of choline in the hippocampus of cLH rats but in contrast to the study by Sartorius et al., there was no effect in wild type control animals (Biedermann et al., 2012). This may be due to normalization of the data or comparison to a baseline scan in Sprague-Dawley control rats. Interestingly, choline MRS signal was also increased in the prefrontal cortex of patients treated with the SSRI paroxetine (Zhang et al., 2015).

Previous studies have examined the effect of early life adversity in the form of maternal separation (MS) on metabolite concentrations, however no difference between MS animals and controls was observed however both environmental enrichment and escitalopram treatment increased NAA levels in the (Hui et al., 2010, 2011).

Myoinositol (mI) levels can be indicative of glial cell function or proliferation and it has been considered a proxy marker of inflammation (Kousi et al., 2013). Preclinical MRS studies have shown increased hippocampal myoinositol levels following both CMS (Hemanth Kumar et al., 2012) and the acute FST (Kim et al., 2010). Similarly, increased mI levels have been observed in the amygdala following chronic psychosocial stress (Grandjean et al., 2016). Together, these data suggest a possible link between glial function and depression pathology.

Increased levels of GABA have been reported in the hippocampus of CMS-offspring exposed to an acute stressor (Huang et al., 2016). *Ex vivo* ^1^H-MRS studies support elevated GABA levels following CMS and restraint stress with increases observed in the anterior cingulate and prefrontal cortex, respectively (Perrine et al., 2014; Drouet et al., 2015) suggesting that altered inhibitory neurotransmission may be involved in the pathophysiology of CMS.

Alterations in the N-acetyl aspartic acid (NAA), a marker commonly used to indicate neuronal integrity have also been reported, with decreased hippocampal NAA levels following both mild and unpredictable chronic stress paradigms (Xi et al., 2011; Hemanth Kumar et al., 2012) as well as chronic exposure to the FST (Li et al., 2008). Xi et al. (2011) report that hippocampal NAA reductions were normalized by chronic escitalopram treatment (4 weeks). Conversely, Han et al. (2015) showed increased NAA in both the amygdala and hippocampus following a single prolonged stress. This may be due to single vs. repeated stress protocols used. In addition, MRS studies in three mouse lines bred for HPA axis stress reactivity have revealed decreased N-acetylaspartate (NAA) levels in the dorsal hippocampus and prefrontal cortex which is coupled with decreased levels of performance in hippocampal dependent spatial memory tasks when compared with mice with low HPA axis stress reactivity (Knapman et al., 2012).

It is worth noting that an advantage of animal models is that metabolite concentrations in can also be assessed through invasive or post-mortem methods such as *in vivo* microdialysis or *ex vivo* assessment of tissue concentrations. These techniques could be used to compliment or validate *in vivo* MRS findings.

Results from both preclinical and clinical MRS studies implicate altered glutamatergic signaling in depression with studies in both depressed patients and animal models reporting decreased glutamate and/or glutamine signal, particularly in the prefrontal cortex (Yildiz-Yesiloglu and Ankerst, 2006; Li et al., 2008; Walter et al., 2009; Hemanth Kumar et al., 2012). Clinical MRS results for other metabolites are inconsistent and depend upon subregion investigated, disease duration, depression subtype, and medication (Yildiz-Yesiloglu and Ankerst, 2006) however some studies report increased choline signal in the frontal cortex (Steingard et al., 2000; Farchione et al., 2002), which is also shown in animal models (Han et al., 2015; Grandjean et al., 2016). Interestingly, reductions in the hippocampal glutamate signal differentiated susceptible from resilient animals when exposed to CMS (Delgado y Palacios et al., 2011) and similar alterations have been shown in depressed patients (Block et al., 2009). Alterations in certain metabolite concentrations in preclinical models could be validated through *in vivo* microdialysis or *ex vivo* assessment of tissue concentrations.

#### Resting-state functional MRI (rs-fMRI)

Functional MRI (fMRI) provides a tool to map regional changes in cerebral blood flow at resting state, i.e., in the absence of task execution. Depressed patients have been reported to have decreased dorsolateral prefrontal cortex activation (Alcaro et al., 2010) as well as increased baseline activity in the orbitofrontal and subgenual anterior cingulate cortices (for review see Hasler and Northoff, 2011) relative to controls. Increased resting state perfusion as assessed via arterial spin labeling (ASL) in the subgenual anterior cingulate cortex has also been shown specifically in treatment resistant depressed patients relative to controls (Duhameau et al., 2010). These alterations are sensitive to treatment: resting state perfusion as assessed via ASL in the anterior cingulate cortex, for example, was reduced (Clark et al., 2006) and dorsomedial prefrontal cortical activity was increased following chronic venlafaxine and fluoxetine treatment in depressed patients (Savitz and Drevets, 2009). Hence, unlike some of the structural alterations discussed above, brain functional alterations appear to be state dependent markers of MDD. This is potentially unsurprising as quite modest changes in plasticity may induce marked changes in blood flow or neuronal activity however alterations in brain structure at a resolution detectable would be harder to induce.

The default mode network is a set of brain regions which are more active at rest than during task execution, which include the orbital frontal cortex, the medial prefrontal/anterior cingulate cortex, the lateral temporal cortex, the inferior parietal lobe, the posterior cingulate and retrosplenial cortex, the hippocampus and parahippocampal cortex (Lu et al., 2012). Hyperactivity in the default mode network during task performance in depressed patients has been reported and interpreted as a possible MRI marker for rumination (for review see Whitfield-Gabrieli and Ford, 2012). Hyper connectivity between the subgenual anterior cingulate and default mode network at rest has also been observed in MDD (Greicius et al., 2007; Berman et al., 2011), and increased default mode network connectivity with the dorsolateral prefrontal cortex are thought to underlie some aspects of emotional dysregulation characteristic of the disorder (Sheline et al., 2010).

Preclinical studies have begun to employ fMRI to investigate regional activity and functional connectivity *in vivo* and extraction of the blood oxygen level dependent (BOLD) signal is commonly used as surrogate marker of neuronal activation. Recent studies have shown altered BOLD signal intensity in several brain regions involved in mood regulation following exposure to early-life adversity or stress. Hui et al. (2010) recently showed that animals subjected to the early life maternal separation protocol exhibited increased BOLD signal in the insular lobe, hypothalamus, limbic system, hippocampus, and frontal lobe in adulthood. Similarly, increased BOLD activation was observed in the hippocampus, limbic system and temporal lobe of rats exposed to CMS.

Recent advances in preclinical neuroimaging have allowed the identification of similar resting-state networks in rodents and humans (Lu et al., 2012; Sierakowiak et al., 2015; Zerbi et al., 2015; Gozzi and Schwarz, 2016) and the analysis of these networks in disease models.

Grandjean et al. have shown that chronic psychosocial stress increased within-network functional connectivity in the cingulate cortex and sensory cortical networks, specifically the supplementary, barrel field 1 and 2, and visual cortices in mice (Grandjean et al., 2016). In addition there was also increased between-network functional connectivity between the prefrontal cortex and both the amygdala and piriform cortex; ventral hippocampus and the amygdala and between the cingulate cortex and both the piriform cortex and the amygdala following psychosocial stress. Rodents have also been shown to have a default mode network similar to that seen in humans (Lu et al., 2012) and 10 days of immobilization stress has been reported to increase resting state functional connectivity within this network in addition to the visual and somatosensory networks (Henckens et al., 2015). Altered functional connectivity has also been shown in the congenital learned helplessness rat with increased correlation between activity in the DRN and the somatosensory cortex, orbital cortex, frontal cortex, and caudate-putamen. Enhanced correlations were also observed between the ventral hippocampus and the retrosplenial cortex and caudate putamen and also between the retrosplenial cortex and area 1 of the cingulate cortex (Gass et al., 2014a). Interestingly hippocampal-cingulate connectivity has been positively correlated with depression severity in patients, drawing parallels with the increased hippocampal-retrosplenial connectivity reported in cLH rats. By contrast, there are reductions in inter-hemispheric connectivity, particularly in the sensory, motor, cingulated and infralimbic cortices, the nucleus accumbens and the raphe nucleus of cLH rats (Ben-Shimol et al., 2015). Similarly, the WKY rat has shown increased functional connectivity between the hippocampus and the left frontal association cortex/dorsolateral orbital cortex in a “more immobile” cohort compared to “less immobile” counterparts (Williams et al., 2014). In contrast, hypoconnectivity was observed with the hippocampus and the left somatosensory cortex, the left ventral striatum partially inclusive of the nucleus accumbens core, the bilateral cingulate cortex, the bilateral lateral septum and the left caudate.

A study investigating resting state fMRI in serotonin receptor 1A knockout mice showed reduced functional connectivity between cortical areas (including prefrontal, retrosplenial, and entorhinal cortices) and the hippocampus (mainly CA1 and dentate gyrus, DG). Similarly, reduced functional correlations were observed between the dorsomedial thalamus and both the cortex and hippocampus (Razoux et al., 2013).

**Figure 1:**
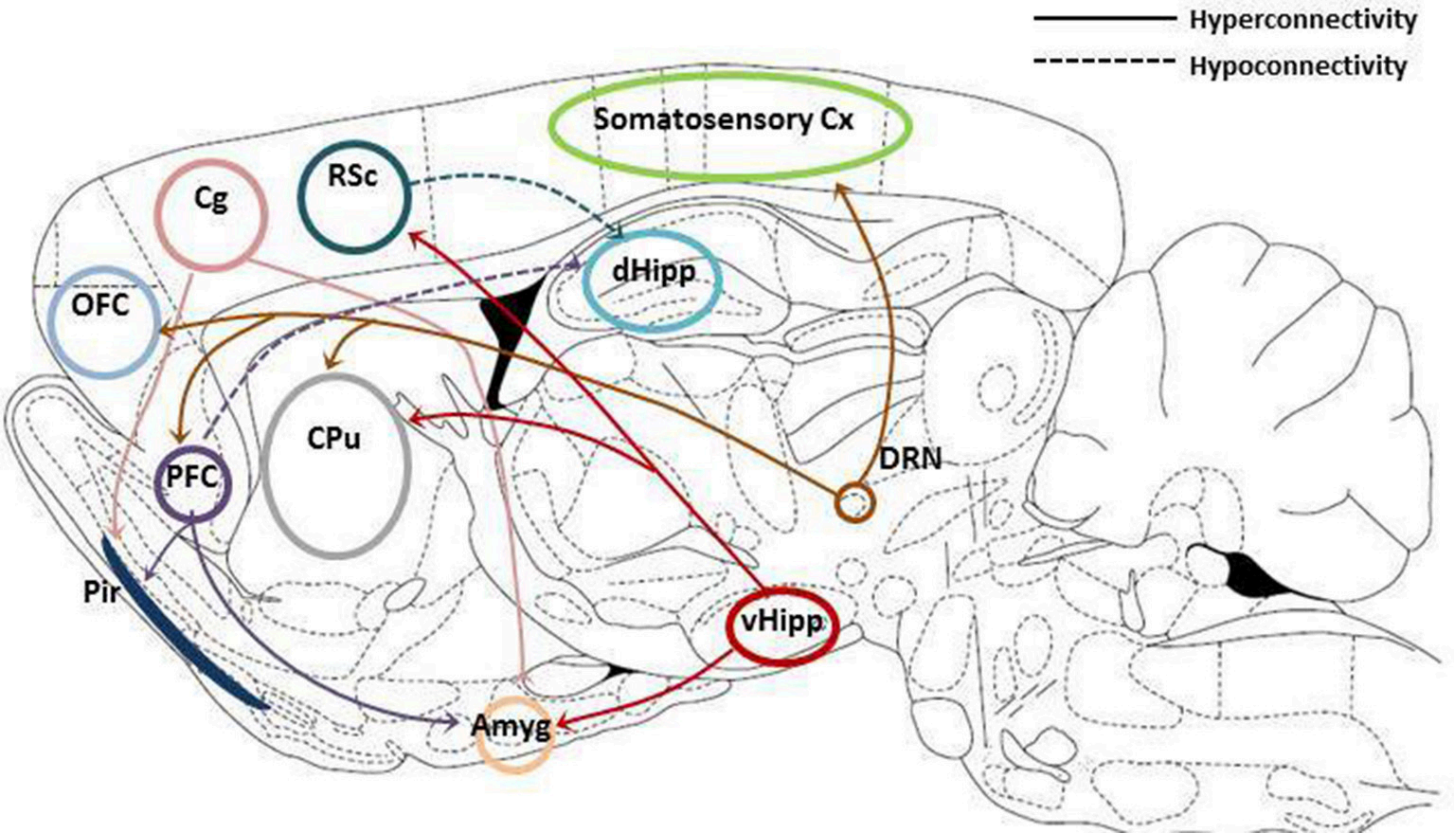
**Summary of resting state functional connectivity changes reported in animal models of depression**. Hyperconnectivity between both the prefrontal (PFC) and cingulated cortices (Cg) and the amygdala (Amyg) and piriform cortex (Pir). Increased connectivity between the ventral hippocampus and the amygdala, retrosplenial cortex (RSc) and caudate putamen (CPu). Hyperconnectivity between the dorsal raphe nucleus (DRN) and the somatosensory cortex (somatosensory cx), the orbirofrontal cortex (OFC), the prefrontal cortex (PFC) and the caudate putamen (CPu). Reduced connectivity has been reported between both the PFC and RSc and the dorsal hippocampus (mainly CA1 and dentate gyrus subregions). Adapted from Paxinos and Watson (1998).

Alterations in functional connectivity reported in animal models of depression are displayed in Figure 1. Studies in both depressed patients and animal models of depression consistently report hyperactivity within the default mode network, comprised of the orbital, prelimbic, auditory/temporal association, parietal, and retrosplenial cortices and the dorsal hippocampus in the rat. In addition, studies in depressed patients have also shown hyperactivity between the cingulate cortex and the amygdala, parts of the affective network, which is also reported in the rodent literature. Interestingly, sub-anaesthetic doses of ketamine increased functional connectivity between frontocortical regions and also the prefrontal cortex and the hippocampus in both rats and humans (Grimm et al., 2015; Becker et al., 2016) suggesting that resting state fMRI may be a valuable translational tool for investigating the actions of novel antidepressants.

#### Manganese-enhanced MRI

Manganese-enhanced MRI (MEMRI) is an emerging neuroimaging technique that exploits the capability of manganese (Mn) ions to enter excitable cells through voltage-gated calcium channels. As Mn ions decrease water longitudinal relaxation time this uptake can be visualized by MRI and as such MEMRI is being increasingly used in preclinical studies to investigate alterations in functional networks. Daducci et al. (2014) showed that chronic administration of interferon alpha to rats, commonly used to model chemotherapy-induced depression, reduced the pituitary volume and also reduced activation in cortical areas, particularly the visual and sensory cortices. Reduced MEMRI signal following manganese injection into the raphe nucleus has been shown in rats exposed to CMS in the substantia nigra, hippocampus, entorhinal cortex and the insular cortex, while increased signal was observed in the medial septal nucleus compared to non-stressed control animals (Gordon and Goelman, 2016) suggesting impaired serotonergic connectivity between the raphe and the substantia nigra and hippocampal areas.

#### Cerebral perfusion

High resolution MRI has revealed discrete reductions in regional cerebral blood volume (rCBV) in the left habenula, particularly the lateral area, and increased rCBV in the bed nucleus of the stria terminalis (BNST) in the congenital learned helplessness (cLH) model of depression (Gass et al., 2014a). Altered cerebral blood perfusion in preclinical models of depression has also been measured by bolus-tracking arterial spin labeling (bt-ASL). This non-invasive method directly assesses blood perfusion and provides quantitative measures relating to blood flow. Decreased cerebral perfusion was observed in the striatum and pre-limbic cortex of WKY rats compared to Wistar controls and interestingly, cerebral perfusion correlated with GFAP-positive cell number such that increased GFAP expression corresponded with increased cerebral perfusion (Gormley et al., 2016). In contrast no alterations in regional cerebral perfusion were reported in the OB rat model of depression (Gigliucci et al., 2014), again highlighting that many MR markers associated with depressed phenotypes may be “model” or patient specific.

#### PharmacofMRI

Pharmacological MRI is becoming a more widely used tool in both preclinical and clinical drug research (Jonckers et al., 2015). Insights gained from clinical MRI studies are frequently confounded by illness, chronicity and medication use when attempting to determine if antidepressant drug treatment may influence gray matter volumes or functional MR parameters. PharmacoMRI coupled with *in vivo* structural MRI in animal models represents an approach which allows for effects of acute and chronic drug treatment and subsequent withdrawal, with clinically relevant dosing to be determined on brain structure and function. Findings may then be further evaluated and confirmed in the post-mortem brain using stereological histochemistry. Vernon et al. (2012) reported that chronic lithium treatment in rats induced an increase in whole-brain volume and cortical gray matter without a significant effect on striatal volume. Increased total brain volumes were subsequently confirmed post-mortem. Comparisons with the antipsychotic haloperidol indicated that the distribution of changes was topographically distinct.

Reductions in brain activation are observed with phMRI following acute treatment with the serotonin (5-HT) reuptake inhibitor (SSRI) fluoxetine (10 mg/kg oral or 5 mg/kg i.v.) in rats (Bouet et al., 2012). Subsequently Klomp et al. (2012) employed pharmacoMRI to assess the effects of chronic treatment with fluoxetine (5 mg/kg orally for 3 weeks) in juvenile (post natal day 25) and adult rats (post natal day 65) following a 1 week washout, using an acute fluoxetine challenge (5 mg/kg i.v.) to trigger the serotonergic system. A significant age by treatment interaction was observed in several subcortical brain regions related to 5-HT neurotransmission, suggestive of differential neuronal effects of SSRI treatment in juveniles that may underlie emotional disturbances seen in adolescents treated with fluoxetine. Harris and Reynell (2016), using data derived from animal studies, consider the underlying mechanisms of antidepressant treatment-related changes in BOLD including alterations in neurovascular coupling and brain energetics and metabolism.

To aid interpretation of spatially distributed activation patterns in response to pharmacological stimuli, Bruns et al. (2015) have proposed a set of multivariate metrics termed domain gauges which are calibrated based on different classes of reference drugs including antidepressants. The profile provides quantitative activation patterns with high biological plausibility with the potential to be developed as a valuable analytical tool for interpretation and decision making in drug development.

A number of recent investigations have assessed the effects of sub-anaesthetic doses of ketamine on intrinsic BOLD connectivity within hippocampal-prefrontal circuits in the rat (Gass et al., 2014b; Becker et al., 2016). Hippocampal-prefrontal cortical connectivity is a key substrate hypothesized to be associated with cognitive and emotional state in central nervous system disorders. Ketamine, a NMDA receptor antagonist, is a psychomimetic agent and rapidly acting antidepressant in the clinic (Zarate et al., 2006) and thus understanding its acute modulatory effect on functional connectivity in the rat brain using rs-fMRI is of significant interest. Ketamine provokes a dose dependent increase in prefrontal connectivity (between the posterior hippocampus, retrosplenial cortex, and prefrontal regions) and these changes are highly concordant and directionally consistent with network reconfigurations observed in humans (Becker et al., 2016). A recent investigation of network reconfiguration induced by ketamine in anaesthetized monkeys assessed differences in functional networks 18 h after drug administration. Here, a down regulation most prominently in the orbital prefrontal cortex, the subgenual and posterior cingulate cortices and the nucleus accumbens were reported (Lv et al., 2016). The changes were reported to oppose the maladaptive alterations characteristic in the depressed brain and further suggested to reflect alterations in local synaptic plasticity triggered by blockade of NMDA receptors leading to network reconfiguration within cortico-limbic circuits. The technique may therefore serve as a novel approach for the identification of translational imaging biomarkers in drug development.

## Limitations of preclinical imaging

A limitation in the interpretation of animal MRI results is that animals are usually anaesthetized when undergoing MRI scans which may alter resting state brain function and perfusion. This is particularly important when considering the interpretation of resting state functional connectivity studies, however the choice of anaesthetic is important in this regard with α2-adrenoceptor agonist medetomidine shown to be better suited to resting state functional connectivity studies than inhalational volatile anaesthetics, such as isoflurane (Grandjean et al., 2014; Nasrallah et al., 2014). For most other imaging acquisitions isoflurane is still the most commonly used anaesthetic (for review see Hanusch et al., 2007; Hoyer et al., 2014). As always the size of the rodent brain makes high resolution imaging, and obtaining adequate signal-to-noise ratios challenging, particularly in relation to MRS. However, with the increasing field strength of preclinical scanners and also advanced coil systems these issues are being overcome.

In addition, an advantage of preclinical MRI is the ability to couple translational neuroimaging techniques with invasive or post-mortem investigations of molecular or cellular alterations associated with the phenotypes. Currently very few preclinical MRI studies incorporate significant post-mortem investigations and including invasive or *ex vivo* techniques such as immunohistochemistry for cell markers, or measurement of analytes in brain regions would advance interpretation of MRI findings.

Furthermore, the most common analysis methods for preclinical data rely upon human software packages which are not designed for the size and composition of the rodent brain therefore much of the pre-processing needs to be done manually, which whilst being labor intensive also introduces some investigator bias. There are ways to make rodent data compatible with human packages, with in-house scripts and some preclinical packages being developed and made freely available (e.g., DBAPI, SPM Mouse), and although progress is being made (Ioanas et al., 2017), a custom built, user friendly analysis package for both structural and functional preclinical MRI analysis would enable preclinical neuroimaging to be more accessible, therefore increasing the scope of the field.

Finally, it is worth highlighting that one of the benefits of animal models is the ability to use invasive techniques and given the limitations described above, preclinical MRI may not always be the best tool for delineating certain aspects of the pathophysiology. Tools such as optogenetics (Touriño et al., 2013) and positon emission tomography (PET) have allowed dissection of the neural circuits involved and alterations in neurochemistry related to depressive symptoms. Perhaps using these techniques in combination with translational MRI will further elucidate the functional alterations in depression and mechanism of antidepressant action.

## Conclusions

This review describes the recent findings in preclinical neuroimaging in animal models of stress and depression and highlights modalities that are particularly promising in translating results from animal models to clinical studies. Advances in preclinical models of depression with MRI-related changes reflecting the human condition, will further our understanding of the pathophysiology of MDD, and potentially uncover novel opportunities for drug development. Furthermore, this review emphasizes the potential for multi-modal MRI as a tool to highlight detectable physiological markers which may be useful indicators or susceptibility and lead to the development of clinical strategies that allow for patient stratification and tailored therapeutic strategies.

## Author contributions

AM and SG prepared an initial draft of the animal section and LT prepared an initial draft of the clinical section of the manuscript. TF and AH contributed to writing all sections, and oversaw revisions and preparation of the final manuscript.

### Conflict of interest statement

The authors declare that the research was conducted in the absence of any commercial or financial relationships that could be construed as a potential conflict of interest. The reviewer BS and handling Editor declared their shared affiliation, and the handling Editor states that the process nevertheless met the standards of a fair and objective review
